# Onset of Late Cretaceous diversification in Europe’s freshwater gastropod fauna links to global climatic and biotic events

**DOI:** 10.1038/s41598-022-06557-1

**Published:** 2022-02-17

**Authors:** Thomas A. Neubauer, Mathias Harzhauser

**Affiliations:** 1 Department of Animal Ecology and Systematics, Justus Liebig University, Heinrich-Buff-Ring 26 (iFZ), 35392 Giessen, Germany; 2 Naturalis Biodiversity Center, Leiden, The Netherlands; 3 Geological-Paleontological Department, Natural History Museum Vienna, Vienna, Austria

**Keywords:** Palaeontology, Speciation

## Abstract

The Mesozoic rise of the European freshwater gastropod fauna is still poorly understood. Compared to the well documented Cenozoic history, little is known about the patterns and processes underlying the early diversification preceding their extinction crisis at the K–Pg boundary. We assess what is probably a first pulse of diversification of the Cenozoic-type fauna in the Late Cretaceous along with the potential abiotic and biotic controls for shifts in species diversification. We find strong support that the increase in the speciation rate in the Santonian (~ 85 Myr ago) is linked to a global sea level rise, which caused extensive flooding of continental areas and the formation of vast brackish-water ecosystems. The following decline of the speciation rate coincides with a rise in diversity and reflects increasing interspecific competition. The peak in the speciation rate postdates the Cenomanian–Turonian Thermal Maximum, which probably limited the potential for diversification among freshwater gastropods due to ecological constraints. The peak coincides moreover with the end phase of the Cretaceous Terrestrial Revolution, which sparked the radiation of angiosperms. The expansion and diversification of flowering plants, being an important food source for freshwater gastropods today, could have formed a necessary basis for gastropod diversification.

## Introduction

Understanding the processes that lead to patterns of species diversity is a focal research topic today. A crucial aspect is concerned with the abiotic and biotic controls of species diversification, i.e., the origination (speciation) and disappearance (extinction) of species through geological time^1–7^. Shifts in climate, habitat type and availability, biotic interactions, and evolutionary innovations are but a few of the commonly found drivers of deep-time diversification^5^.

The majority of studies focus on marine or terrestrial biota, while freshwater organisms, and particularly freshwater invertebrates, have received comparatively little attention. Today, invertebrates make up approximately 85% of species diversity in freshwater environments^8^. Understanding the controls of species diversification in the fossil record is essential to better understand biodiversity change in freshwater ecosystems. By establishing natural baseline rates and assessing the magnitude and rate change that may be considered natural we can disentangle natural and anthropogenic impact on species diversification.

In a previous study, we investigated the diversification history of European freshwater gastropods, with specific focus on the K–Pg boundary event^9^. That study identified a sharp rise in species diversity in the Late Cretaceous (~ 85 Myr ago) following a long interval of low diversity during the early–mid Cretaceous. This increase coincided with a peak in the speciation rate, which rose to about ten times above the normal background rate. We hypothesize that this event represents a first major pulse in the diversification history of Europe’s freshwater fauna, which was abruptly stalled by the K–Pg boundary event. However, the causes for this diversification event are not understood.

Here, we investigate potential controls leading to that speciation peak in the Late Cretaceous. We also estimate species diversity with a recently developed method based on diversification rates that accounts for sampling and preservation heterogeneity through time. We discuss the findings in the context of Europe’s paleogeographic and paleoclimatic development as well as coeval biotic events in other species groups.

## Results

**Figure 1:**
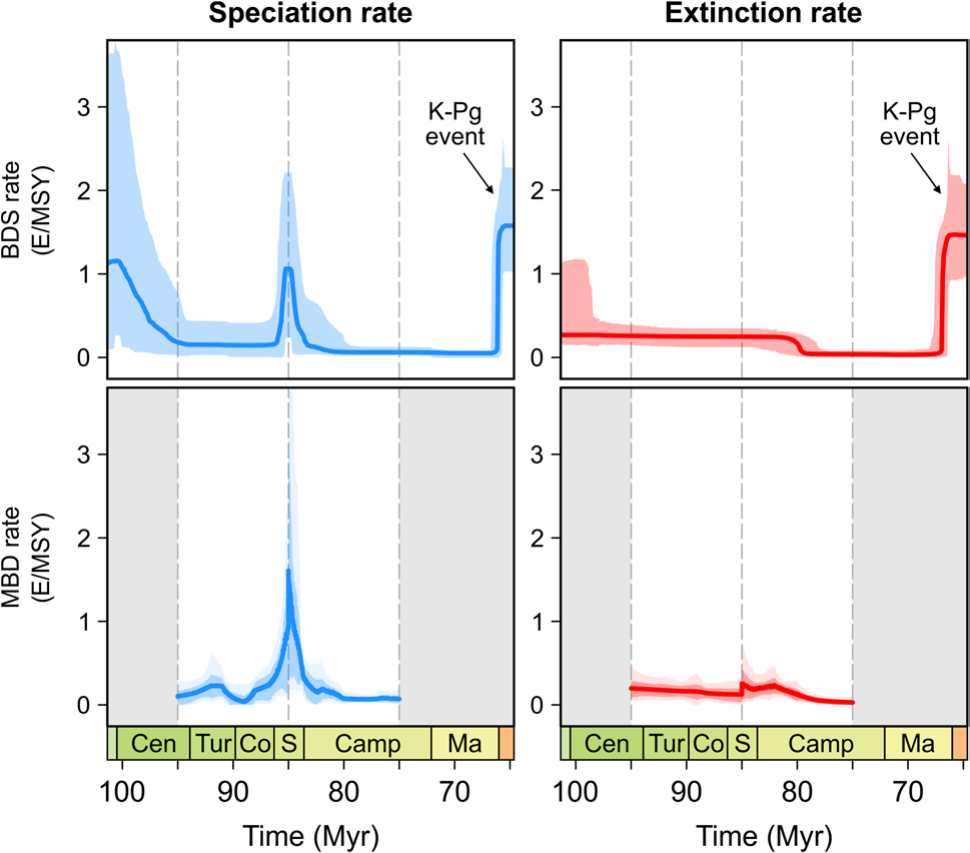
Median diversification rates for the birth–death model with shifts (BDS; after Ref.^9^) and the multivariate birth–death model (MBD) for the 95–85 Myr and 85–75 Myr windows, demonstrating a good match between both models. See Supplementary Fig. S2 for the complete Jurassic–Pleistocene BDS rates.

The speciation and extinction rates inferred by the multivariate birth–death (MBD) analyses as a function of the abiotic and biotic factors yielded a good match with the original rates inferred with the birth–death model with shifts (BDS) (Fig. 1). The timing and magnitude of the speciation peak 85 Myr ago is well captured; only its onset in the MBD analyses slightly predates the actual rise. The plot for the extinction rate (Fig. 1) indicates a minor shift 85 Myr ago, which is however the result of combining two independent analyses and does not reflect a real event. The overall good match suggests that the set of factors chosen in this study accounts well for the observed shifts in diversification.

**Figure 2:**
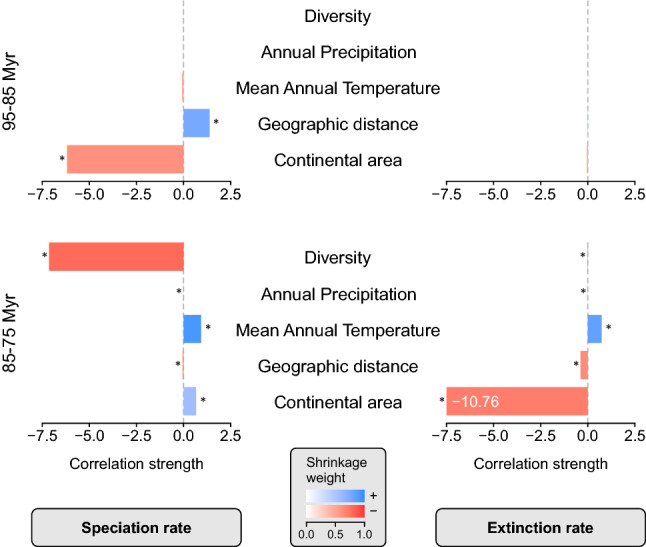
Correlation strengths of abiotic and biotic factors for the two intervals before and after the peak in the speciation rate 85 Myr ago. Asterisks indicate significant correlations (mean shrinkage weight > 0.5). Note the bar for the association between continental area and extinction rate in the 85–75 Myr window is truncated for display purposes.

The rise of the speciation rate in the interval 95–85 Myr correlates significantly with continental area and geographic distance (Fig. 2). The extinction rate is constant throughout that time interval, which is why the lack of a correlation with any biotic or abiotic factor is not surprising. All factors contribute significantly to the decrease of the speciation rate between 85 and 75 Myr ago, but only the negative association with diversity has a notable correlation strength. The minor drop in the extinction rate strongly correlates negatively with area; similar to the speciation rate, all other correlations are significant but minor (Fig. 2).

**Figure 3:**
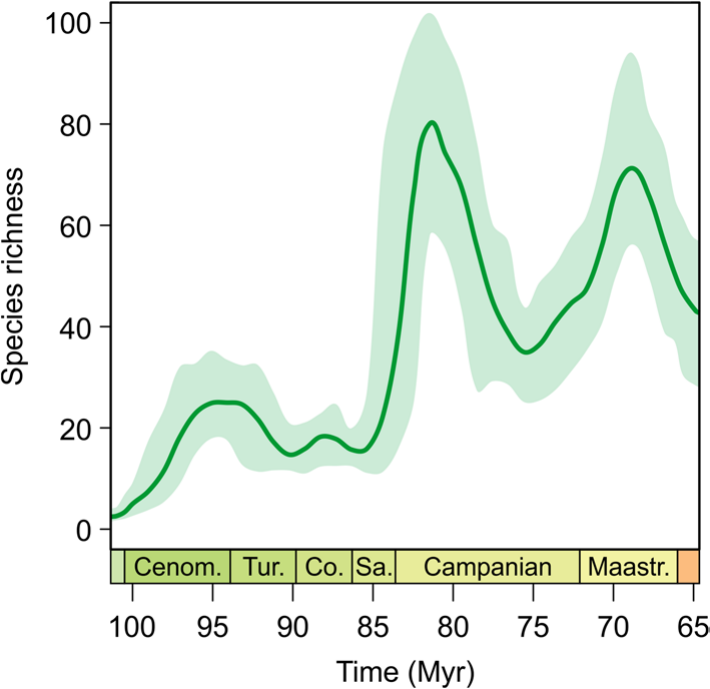
Reconstructed species richness for Late Cretaceous European freshwater gastropods. See Supplementary Fig. S3 for the complete Jurassic–Pleistocene diversity trajectory.

The corrected diversity trajectory reconstructed from the diversification rates indicates a major increase in species richness following the speciation peak 85 Myr ago (Fig. 3). Diversity peaks around 81 Myr ago in the early Campanian and drops again towards the late Campanian.

## Discussion

### Sea level change as a driver of diversification

The peak in the speciation rate in European freshwater gastropods 85 Myr ago is a result of both biotic and abiotic factors. While the rise in speciation is controlled by decreasing continental area (corresponding to an increasing sea level) and, to a lesser extent, increasing geographic distance, its decline is governed by the simultaneous increase in diversity (Fig. 3). The minor decrease in the extinction rate in the Campanian (c. 80 Myr ago) is mainly driven by an increase in Europe’s continental area (Fig. 3).

**Figure 4:**
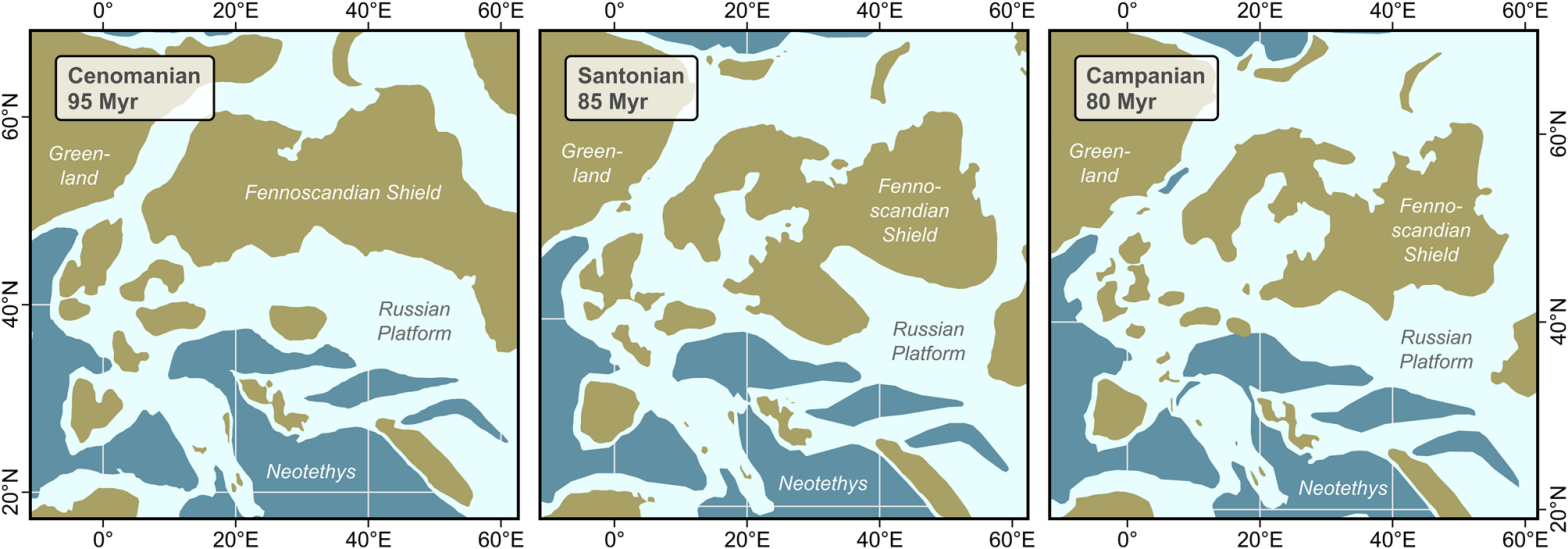
Paleogeographic reconstructions for the interval 95–80 Myr ago, based on Ref.^35^. Light blue areas delimit continental shelf margins (− 1400 m)^35^. Map was generated in R v. 4.0.3 (https://www.r-project.org/).

The shrinking continental area is the result of global sea level rise^10^, which likely came with a shift in the ecosystems available to the freshwater gastropods. The sea level rose by several meters between the late Coniacian and early Santonian^10^, which probably produced extensively flooded areas and fresh- to brackish-water embayments^11^ (Fig. 4). This interpretation is supported by the fact that many diverse faunas of that time across many different countries are found in brackish-water deposits and/or contain a mix of marine, brackish, and continental faunas^12–16^. Similar patterns are found among the fish fauna of that time^17^. Possibly, the extensive brackish-water zone provided the ideal habitat where species could evade competition with marine faunas.

The positive weak correlation between the rising speciation rate and geographic distance suggests that faunas distributed over a larger geographic region are more likely to spawn new species. This result confirms earlier hypotheses that larger areas, i.e., more habitable space, promotes speciation^18^. Additionally, we hypothesize that interspecific competition, which could otherwise constrain diversification and lower the speciation rate^19^, may be avoided more effectively across larger areas.

The Santonian–Campanian decline of the speciation rate (c. 85–80 Myr) is controlled almost exclusively by the coeval rise in diversity (Figs. 1, 2, 3), which likely increased interspecific competition, which in turn slowed down diversification—a text-book example for diversity dependence^19^. The negative association between the extinction rate and area for that time window (85–75 Myr) indicates that the (on average) falling global sea level during the Campanian^10^ lowered the extinction rate. Perhaps, the sea level drop coincided with an increase in freshwater ecosystems and habitable space, which in turn allowed species to distribute over large areas (which is also indicated by the weak negative correlation with geographic distance) and limited the risk of extinction. However, considering the only weak trend in the extinction rate, the biological significance of this association is questionable and the high correlation strength should not be overinterpreted.

### Why did freshwater gastropods diversify in the Late Cretaceous?

While the factors driving and constraining the speciation rate are well understood, the reason why the gastropods radiated exactly at that time and not earlier is not. Many of the modern gastropod families already existed at that time; only the Melanopsidae originated during the speciation event^20^. Instead, diversification happened primarily on the species and genus levels. We do not expect a strong bias of the results from limited sampling^21^, since the BDS model specifically accounts for sampling and preservation heterogeneity. Also, our dataset contains fresh- to brackish-water faunas throughout the Late Cretaceous, with several faunas preceding and postdating our study interval. Therefore, limited sampling could only have an effect on the precise timing and magnitude of the observed peak.

The speciation peak coincided with the final phase of the Cretaceous Terrestrial Revolution (~ 125–80 Myr ago), featuring the coeval radiation of angiosperms, insects, reptiles, birds, and mammals^22,23^. Angiosperms provide an important food source for gastropods today^24^. Especially in the absence of diatoms, which constitute another important food resource^24^ but only entered freshwater ecosystems in the latest Cretaceous^25^, the radiation and expansion of angiosperms may have formed an important basis for the diversification of freshwater gastropods.

Moreover, the speciation peak follows one of the hottest periods in Earth history, the Cenomanian–Turonian Thermal Maximum^26,27^. Average global temperature climbed to ~ 28 °C and sea surface temperatures rose to as much as 38 °C^26,27^. Marine animal diversity experienced a temporary peak during that time^5,28^. Many extant European freshwater gastropods survive water temperatures > 30 °C^29^. However, such high temperatures coincide with lower concentrations of dissolved oxygen, which impacts on the survival and fecundity of freshwater snails^30^. Hence, hothouse conditions like during the Cenomanian–Turonian are unlikely to trigger species extinctions on a larger scale, but the increased extrinsic stress may have constrained their potential for speciation.

## Methods

### Fossil occurrence data and diversification analyses

The underlying data and initial diversification analyses derive from Ref.^9^ (see Supplementary Figs. S1–2). The dataset comprising the entire fossil record of European freshwater gastropods from the Jurassic to the Pleistocene was assembled from the literature and carefully curated. Latest systematic revisions were considered to account for recent changes in synonyms and systematic rank, and uncertain taxonomic records were excluded. Fully freshwater as well as oligohaline taxa were included, mesohaline species were excluded. The most recent stratigraphic concepts for lithostratigraphic and biostratigraphic units were adopted to ascertain correct age correlation. The final dataset includes 24,759 occurrences, 3122 species and 5564 localities (for more details on the taxonomic, stratigraphic, and geographic selection criteria see Ref.^9^).

We employed a two-step birth–death model with shifts (BDS)^1^ based on 200 randomized datasets, where ages were resampled within the respective temporal range^31^. Analyses were performed with the open-source program PyRate (https://github.com/dsilvestro/PyRate), which models fossil occurrences as the combined result of speciation, extinction, and preservation rates. Species diversification was inferred using a Reversible Jump Markov Chain Monte Carlo (RJ-MCMC) algorithm, where rates are allowed to shift over time^31^. In a first step, posterior estimates on speciation and extinction times were obtained by running analyses with 200 million MCMC generations with a sampling frequency of 20,000, discarding the first 20% as burn-in. Three posterior samples of speciation and extinction times were extracted from each resulting log-file. The number of rate shifts were inferred from the resulting 600 replicates in a second round of analyses. Effective sampling sizes were assessed with the ‘coda’ package v. 0.19–361^32^ for the statistical programming environment R v. 3.6.3^33^. The subsequent multivariate analyses were based on the 100 log-files with highest effective sampling sizes.

### Estimating controls of diversification

We employed the multivariate birth–death (MBD) model developed by Ref.^2^ using a set of four abiotic and one biotic predictor variables to identify the factors controlling the speciation peak 85 Myr ago. The abiotic variables included in this study comprise one geographic (continental area), one biogeographic (geographic distance among localities), and two climatic factors (regional temperature and precipitation). Europe’s continental area was estimated as a proxy for the effect of global sea level variation, which may be a more useful estimator for terrestrial ecosystems than absolute sea level data. Global sea level shifted considerably throughout the Cretaceous^10^, which may have affected the brackish-water to freshwater habitats along Europe’s coastline. Geographic distance, quantified as minimum spanning tree distance among all localities of a given time slice, was included as a measure for biogeographic isolation of faunas. Temperature and precipitation have been found important controls of species diversity and diversification in previous studies^5,34^. Details on the quantification of these variables can be found in the Supplementary Methods. Diversity is included as biotic factor per default in the MBD model, which automatically includes a correction for preservation and sampling heterogeneity (see previous chapter).

We lately demonstrated the importance of a time-varying perspective of the controls of diversification^34^. Too short or too long time intervals may yield only poor or inconclusive results, since they can confound different processes leading to different shifts in diversification rates. In order to disentangle the factors that contribute to the increase and the decrease in the speciation rate, respectively, we selected two 10 Myr windows—one before (95–85 Myr) and one after the peak (85–75 Myr). Apart from the decrease in the extinction rate c. 80 Myr ago, these windows do not contain other significant shifts. Thereby, we avoid a potential bias of a correlation with other events.

For each of the two windows, 100 replicate MBD analyses were executed using the rescaling option to scale factors to a comparable range. Analyses were run for 40 million MCMC generations at a sampling frequency of 50,000, discarding the first 20% as burn-in. We calculated the median correlation strengths (instead of the mean as in Ref.^2^) to avoid a bias of outliers and the mean shrinkage weights across the 100 replicates. Mean shrinkage weights > 0.5 are considered to reflect significant correlations^2^.

### Estimating true diversity

To estimate true diversity, we employed the mcmcDivE algorithm, which is part of the PyRate v. 3 package developed by D. Silvestro (https://github.com/dsilvestro/PyRate/tree/master/mcmcDivE). This method allows correcting for the preservation bias, which supposedly increases with time^31^. Preservation is estimated directly from the BDS model and independently for each geological epoch. Diversity was estimated for 200 equal-size time bins, which yields a resolution of approximately 1 Myr across the Jurassic–Pleistocene time frame of the BDS analyses. Considering the huge data amount resulting from BDS analyses, 100 random generations were extracted from each of the final 100 MCMC log-files. The analysis was run for each of the hundred replicates for 2 million MCMC iterations with a sampling frequency of 1,000 to achieve reasonably high effective sampling sizes (> 100).

## Supplementary Information

Supplementary Information.

## Data Availability

The data that support the findings of this study are available in the supplementary material of this article. The dataset is available at http://dx.doi.org/10.22029/jlupub-9.
