# Supplementary material for: Onset of Late Cretaceous diversification in Europe’s freshwater gastropod fauna links to global climatic and biotic events
Source: Sci Rep. 2022 Feb 17;12:2684. doi: 10.1038/s41598-022-06557-1 (PMC8854554; doi:10.1038/s41598-022-06557-1)
Supplement: Supplementary file 1 — Supplementary Information. [file 41598_2022_6557_MOESM1_ESM.pdf]

Onset of Late Cretaceous diversification in Europe's freshwater gastropod fauna links to global climatic and biotic events

Supplementary information

Thomas A. Neubauer[1,2] & Mathias Harzhauser[3]

[1]Department of Animal Ecology and Systematics, Justus Liebig University, Giessen, Germany

[2]Naturalis Biodiversity Center, Leiden, The Netherlands

[3]Geological-Paleontological Department, Natural History Museum Vienna, Vienna, Austria

This file contains:

- Supplementary Methods, including details on the quantification of abiotic variables used in the analyses
- Supplementary Figures S1–S3

Supplementary Methods

Quantification of abiotic variables. Continental areas of Europe through the Late Cretaceous were reconstructed based on the latest paleogeographic maps for the Cenomanian to Maastrichtian by ref. [1]. For each map, the mean age of the corresponding stratigraphic stage was applied in accordance with the PALEOMAP Project where the original reconstructions derive from ref. [2]. Throughout the Late Cretaceous, Europe was mostly well delimitated by seaways; temporary land bridges to central Asia during the Cenomanian and Maastrichtian were cropped following the current political border of the European continent. Resulting polygons were projected into an equal-area projection (Behrmann) to calculate areas.

Geographic distance among all localities was quantified using minimum spanning tree distance, which has been demonstrated as a reliable measure for spatially incomplete data, such as provided by the fossil record [3]. Minimum spanning tree distance gives the shortest distance that connects all fossil localities. Each locality was assigned an age based on the mean of the uncertainty range of its stratigraphic classification (e.g., chronostratigraphic stages or absolute age boundaries of lithostratigraphic units). Paleocoordinates of the localities were reconstructed based on the plate tectonic model of ref. [2] via the GPlates online portal (http://portal.gplates.org/service/d3_demo/?view=points). Minimum spanning tree distances were then calculated for Late Cretaceous occurrences based on 1 Myr bins.

Regional temperature was reconstructed based on global temperature raster data on a 1° x 1° spatial resolution available through the PALEOMAP project [4]. The related temperature data are based on the HadleyCM3 paleoclimate simulations of ref. [5], which use estimates of the past concentration of atmospheric CO_2 from ref. [6]. It is well-known that global climate models tend to produce cooler temperatures at higher latitudes, especially for hot house time intervals such as

the Cretaceous. In order to correct this bias, we adjusted the latitudinal temperature gradient using the Phanerozoic climate model of ref. [7]. European average (“regional”) temperature and precipitation per time slice were calculated as the mean across all raster cells that intersect with the polygons created in the previous step.

Area and geographic distance were log10-transformed, and all abiotic factors were interpolated to 0.1 Myr steps. All factor quantification and transformation was done in R v. 4.0.3 [8] using the packages ‘GeoRange’ v. 0.1.0 [9], ‘raster’ v. 3.4-5 [10], ‘rgeos’ v. 0.5-5 [11], and ‘sf’ v. 0.9-7 [12].

Supplementary Figures

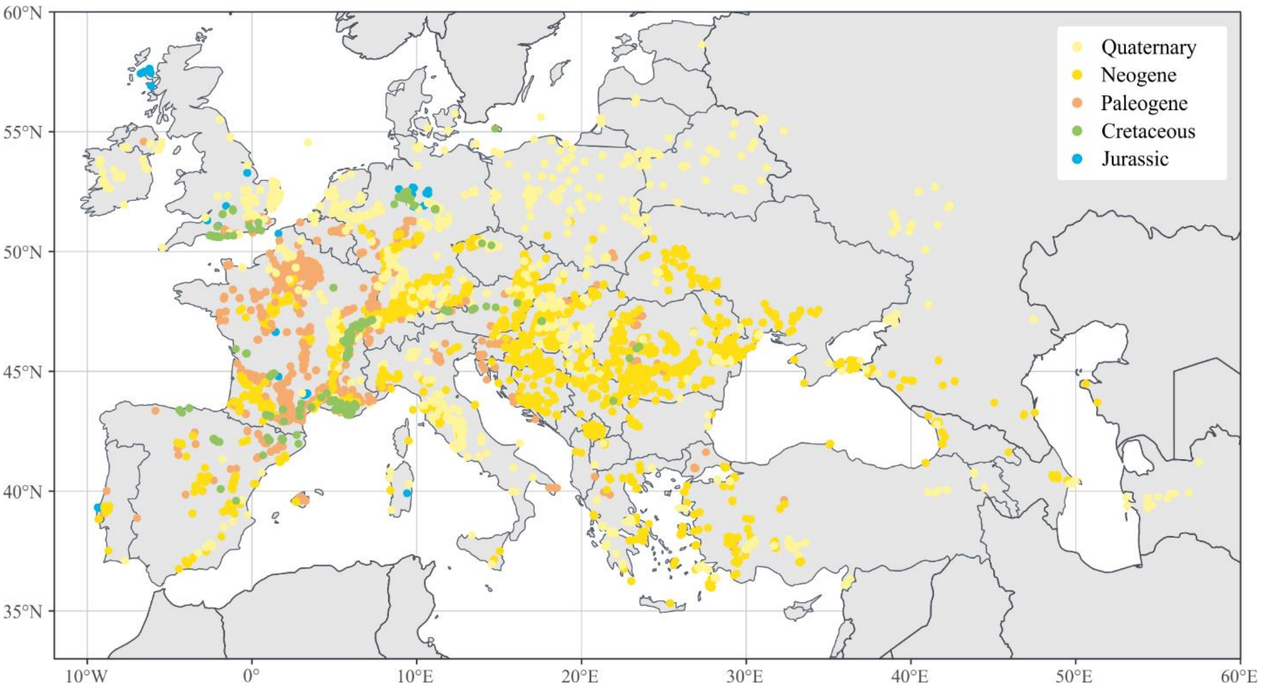

Figure S1. Localities yielding Jurassic to Pleistocene (201.3–0.0117 Myr ago) freshwater gastropods included in the dataset (after ref. [13]). Colour codes conform to the International Chronostratigraphic Chart (see https://stratigraphy.org/chart). Map was generated in R v. 4.0.3 (https://www.r-project.org/).

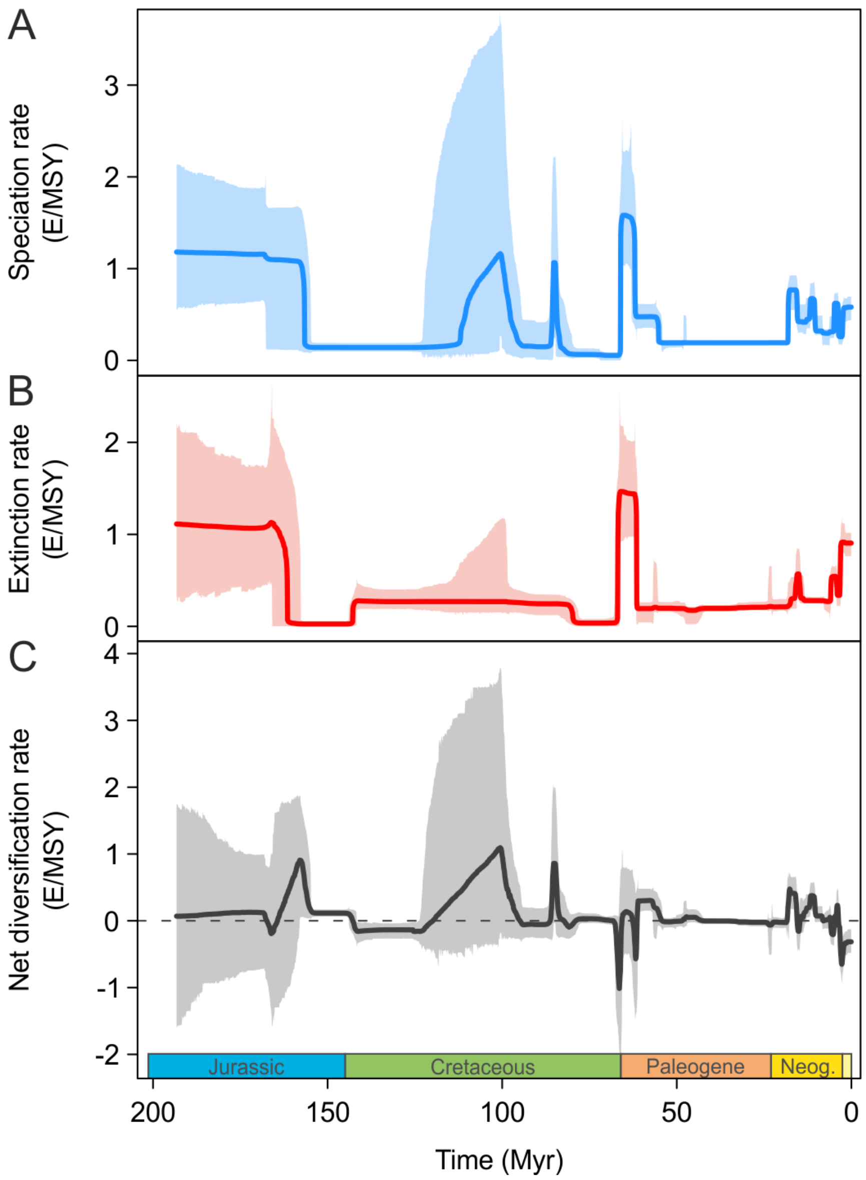

Figure S2. Jurassic–Pleistocene rates of diversification of European freshwater gastropods. (A) Speciation rate. (B) extinction rate. (C) net diversification rate, calculated as speciation minus extinction rate. Shown are the median rates and the 95% highest posterior density quantifying the uncertainty in rates. The figure shows diversification peaks and plateaus preceding our study interval in the Late Cretaceous (Fig. 1), but those are associated with high uncertainties and based on few species and occurrences only. E/MSY, events per million species years.

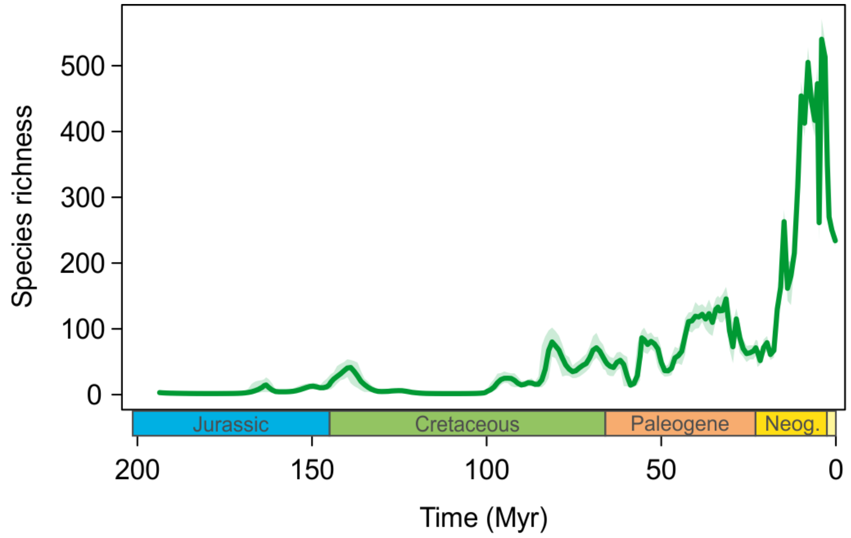

Figure S3. Complete Jurassic–Pleistocene diversity trajectory accounting for sampling and preservation heterogeneity, based on the mcmcDivE algorithm.

References

1. Kocsis, A. T. & Scotese, C. R. Mapping paleocoastlines and continental flooding during the Phanerozoic. *Earth-Sci. Rev.* **213**, 103463 (2021).
2. Scotese, C. R. & Wright, N. PALEOMAP Paleodigital Elevation Models (PaleoDEMS) for the Phanerozoic. PALEOMAP Project, Evanston, IL. https://www.earthbyte.org/paleodem-resource-scotese-and-wright-2018 (2018).
3. Boyle, J. Quantifying geographic range measures and their utility as extinction risk proxies. *PeerJ Preprints* **5**, e3379v1 (2017).
4. Scotese, C. R. An Atlas of Paleogeographic Maps: The Seas Come In and the Seas Go Out. *Annu. Rev. Earth Planet. Sci.* **49**, 669-718 (2021).
5. Valdes, P. J., Scotese, C. R. & Lunt, D. J. Deep ocean temperatures through time. *Clim. Past* **17**, 1483-1506 (2021).
6. Foster, G. L., Royer, D. L. & Lunt, D. J. Future climate forcing potentially without precedent in the last 420 million years. *Nat. Commun.* **8**, 14845 (2017).
7. Scotese, C. R., Song, H., Mills, B. J. W. & van der Meer, D. G. Phanerozoic paleotemperatures: The earth's changing climate during the last 540 million years. *Earth-Sci. Rev.* **215**, 103503 (2021).
8. R Core Team. R: A language and environment for statistical computing. Version 4.0.3. R Foundation for Statistical Computing, Vienna. http://www.R-project.org (2020).
9. Boyle, J. GeoRange: Calculating Geographic Range from Occurrence Data. R package version 0.1.0. http://CRAN.R-project.org/package=GeoRange (2017).
10. Hijmans, R. J. et al. raster: Geographic Data Analysis and Modeling. R package version 3.4-5. https://rspatial.org/raster/ (2020).

11. Bivand, R. et al. rgeos: Interface to Geometry Engine – Open Source ('GEOS'). R package version 0.5-5: https://cran.r-project.org//rgeos/index.html (2020).
12. Pebesma, E. Simple Features for R: Standardized Support for Spatial Vector Data. *R J.* **10**, 439-446 (2018).
13. Neubauer, T. A. et al. Current extinction rate in European freshwater gastropods greatly exceeds that of the late Cretaceous mass extinction. *Commun. Earth Environ.* **2**, 97 (2021).
